# Sensilla-Specific Expression of Odorant Receptors in the Desert Locust *Schistocerca gregaria*

**DOI:** 10.3389/fphys.2019.01052

**Published:** 2019-08-22

**Authors:** Xingcong Jiang, Heinz Breer, Pablo Pregitzer

**Affiliations:** Institute of Physiology, University of Hohenheim, Stuttgart, Germany

**Keywords:** olfaction, insect, desert locust, antenna, odorant receptors, sensilla

## Abstract

The desert locust *Schistocerca gregaria* recognizes multiple chemical cues, which are received by olfactory sensory neurons housed in morphologically identifiable sensilla. The different sensillum types contain olfactory sensory neurons with different physiological specificities, i.e., they respond to different categories of chemical signals. The molecular basis for the sensilla-specific responsiveness of these cells is unknown, but probably based on the endogenous receptor repertoire. To explore this issue, attempts were made to elucidate whether distinct odorant receptors (ORs) may be expressed in a sensilla-specific manner. Analyzing more than 80 OR types concerning for a sensilla-specific expression revealed that the vast majority was found to be expressed in sensilla basiconica; whereas only three OR types were expressed in sensilla trichodea. Within a sensillum unit, even in the multicellular assembly of sensilla basiconica, many of the OR types were expressed in only a single cell, however, a few OR types were found to be expressed in a consortium of cells typically arranged in a cluster of 2–4 cells. The notion that the OR-specific cell clusters are successively formed in the course of development was confirmed by comparing the expression patterns in different nymph stages. The results of this study uncover some novel and unique features of locust olfactory system, which will contribute to unravel the complexity of locust olfaction.

## Introduction

Locusts, like the desert locust *Schistocerca gregaria*, are characterized by a remarkable phase polyphenism and can switch between a solitarious and a gregarious phase (Van Huis et al., 2007; Pener and Simpson, 2009). In the gregarious phase, migrating locust swarms can cause severe agricultural and economic damages in the habituated areas like Africa and Asia (Roffey and Popov, 1968; Skaf et al., 2006; Simpson and Sword, 2008; Pener and Simpson, 2009). The molecular mechanisms underlying the phase transition are under rigorous investigation but still remain poorly understood (Kang et al., 2004; Guo et al., 2011; Rogers et al., 2014; Wang et al., 2014; Yang et al., 2014). Besides tactile and visual stimuli, the sense of smell plays an important role in the life cycle and phase change of locusts (Ould Ely et al., 2006; Cullen et al., 2010; Maeno et al., 2011; Wang et al., 2015, 2019; Pregitzer et al., 2017). Locusts are in fact able to recognize a variety of chemical compounds, including green leaf volatiles and chemical cues for aggregation and oviposition (Torto et al., 1994; Rai et al., 1997; Ochieng’ and Hansson, 1999). The primary organ for sensing such chemical compounds is a pair of antennae, covered by hair-like appendage structures called sensilla. The antennal sensilla have been classified based on morphological criteria as basiconic, trichoid, coeloconic, and chaetic sensilla (Stocker, 1994; Ochieng et al., 1998). Encoding the identities of chemical compounds relies on a variety of olfactory receptors including odorant receptors (ORs) which are localized on the chemosensory membrane of olfactory sensory neurons (OSNs) housed within each sensillum. For the antennae of *S. gregaria* it was recently demonstrated that only the OSNs in s. basiconica and s. trichodea express specific odorant receptors and the olfactory receptor co-receptor (Orco) (Yang et al., 2012; Pregitzer et al., 2017). In fact, a large set of OR genes was found for the migratory locust *Locusta migratoria* (Wang et al., 2014) and for *S. gregaria* as many as 119 OR types were identified through an antennal transcriptomic survey (Pregitzer et al., 2017). So far, there is little information concerning the expression of distinct OR types in either s. basiconica or s. trichodea. This issue is of particular interest in view of the unequal distribution of both sensillum types on the antennae of *Schistocerca gregaria*, which according to the studies of Ochieng et al. (1998) comprises more than 1000 sensilla basiconica each with 20–50 OSNs, compared to about 200 sensilla trichodea each with 1–3 OSNs.

Interestingly, a high number of OSNs as in the locust sensilla basiconica is rarely seen in other insect model species, like flies and noctuid moths, where most of the olfactory responsive sensilla comprise only 2–4 OSNs, similar to s. trichoidea in *S. gregaria* (Sanes and Hildebrand, 1976; Venkatesh and Singh, 1984). For these species, characteristic stereotypical patterns of OR expression have been described, indicating that cells expressing a distinct receptor type are stereotypically arranged with cells, which express a matching receptor type (Hallem et al., 2004; Krieger et al., 2009). It is currently unknown whether similar principles may also be effective for the expression of OR types in the sensilla of *S. gregaria*. Moreover, in view of the multicellular assembly in s. basiconica of *S. gregaria* it is interesting to know, whether a specific OR type is confined to a single cell, analogous to other insect species, or is concomitantly expressed in more than one cell. In the present study we set out to explore the sensilla-specific expression patterns of OR types. Moreover, attempts were made to evaluate whether in the multicellular assembly of s. basiconica, one distinct OR type may be expressed in multiple cells.

## Materials and Methods

### Animals and Tissues Treatment

Adult and nymph stages of *Schistocerca gregaria* were purchased from local suppliers. Antennae were dissected using autoclaved surgical scissors. For RNA extraction, the organs were immediately frozen in liquid nitrogen and stored at −70°C.

### Synthesis of Riboprobes for *in situ* Hybridization

Riboprobe generation was performed as described earlier (Pregitzer et al., 2017). OR sequences cloned into pGEM-T vector (Invitrogen) were used for *in vitro* transcription. The linearized pGEM-T vectors containing desert locust OR sequence fragments were utilized to synthesize antisense riboprobes labeled with either digoxigenin (DIG) or biotin (BIO) using the T7/SP6 RNA transcription system (Roche, Germany).

### *In situ* Hybridization

Antennae of male and female adult *Schistocerca gregaria* locusts were crosscut into two halves, embedded in Tissue-Tek O.C.T. Compound (Sakura Finetek, Alphen aan den Rijn, Netherlands) and used to make 12 μm thick longitudinal sections with a Leica CM3050 S cryostat (Leica Microsystems, Bensheim, Germany) at −21°C. Sections were thaw mounted on Super Frost Plus slides (Menzel, Braunschweig, Germany) and stored at −70°C until use. Sections were taken out from the freezer and immediately transferred into fixation solution (4% paraformaldehyde in 0.1 M NaHCO_3_, pH 9.5) for 22 min at 4°C. Next, sections were washed in 1×PBS (0.85% NaCl, 1.4 mM KH_2_PO_4_, 8 mM Na_2_HPO_4_, pH 7.1) for 1 min, incubated in 0.2 M HCl for 10 min and washed twice in 1×PBS for 2 min each. Then sections were incubated for 10 min in acetylation solution (0.25% acetic anhydride freshly added in 0.1 M triethanolamine) followed by three wash steps in 1×PBS (each wash step lasted 3 min). Sections were incubated in pre-hybridization solution [5×SSC (0.75 M NaCl, 0.075 M sodium citrate, pH 7.0) and 50% formamid] for 15 min at 4°C. Sections were hybridized with digoxigenin- and biotin-labeled probes simultaneously. However, for two-color FISH, 100 μl hybridization solution (50% formamide, 2×SSC, 10% dextran sulfate, 0.2 mg/ml yeast t-RNA, 0.2 mg/ml herring sperm DNA) supplemented with labeled antisense RNA was placed per slide onto the tissue sections. After placing a coverslip, slides were incubated in a humid box (50% formamide) at 60°C overnight. Visualization of labeled probes was performed as described previously (Krieger et al., 2002). In short, digoxigenin-labeled probes were visualized by the anti-digoxigenin alkaline phosphatase-conjugated antibody in combination with the HNPP fluorescent detection set (Roche Diagnostics). Incubation with the anti-digoxigenin alkaline phosphatase-conjugated antibody as well as incubation with the HNPP/Fast Red substrate was conducted overnight at 4°C. For visualization of biotin-labeled probes, the TSA fluorescein system kit (PerkinElmer, Waltham, MA, United States) was used. Incubation of sections with biotin-binding streptavidin conjugated to horse radish peroxidase and incubation with fluoresce in conjugated tyramides were conducted overnight at 4°C.

### Analysis of Antennal Sections by Confocal Microscopy

Antennae used for fluorescence *in situ* hybridization were analyzed on a Zeiss LSM 510 meta laser scanning microscope (Zeiss, Oberkochen, Germany). Confocal image stacks of the red and green fluorescence channels as well as the transmitted-light channel were taken. Image stacks were utilized to generate pictures showing projections or selected optical planes, with the fluorescence and transmitted light channels overlaid or shown separately.

For the analyses in adults between 2 and 4 slides for each OR were analyzed. Each slide harbored 10–20 sections from the antennae of 2–5 animals. An individual antennal section comprised - dependent on the quality of the section - between 3 and 15 antennal segments. Each antennal segment contains multiple basiconic OSN clusters depending on the part of the segment that was sliced; the number ranges from about ten up to several dozen clusters (Yang et al., 2012).

The studies on developmental changes in OSN organization are based on 2 to 4 slides for adults and 5th instar nymphs; for 1st instar nymphs 3 (OR67 and OR8) or 4 slides (OR17, OR29, OR35, and OR110) were analyzed. Slides were examined in two-color FISH experiments following a stringent experimental procedure until clear and convincing fluorescent signals emerged. Observed labeling patterns were documented in LSM images taken from the most convincing antennal areas.

## Results

### Sensilla-Specific Expression of Putative Odorant Receptors

**FIGURE 1:**
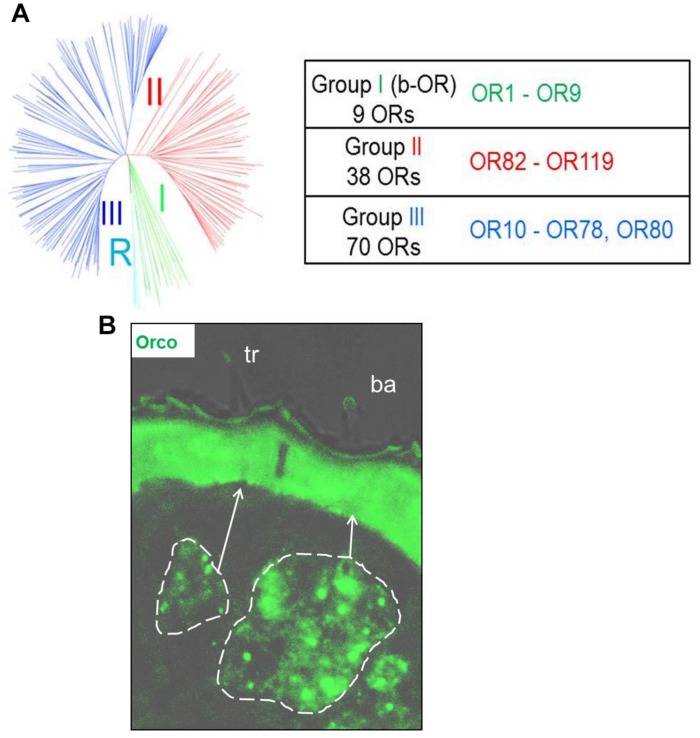
**(A)** The schematic phylogenetic tree of locust OR sequences is adapted from Pregitzer et al., 2017. *Schistocerca* ORs are grouped within 3 distinct monophyletic lineages. Group **I** was previously named b-ORs, due to its close proximity to the basal Orco root group, marked by a R. A monophyletic group diverging from an inner-node in the phylogenetic tree, supported by a bootstrap value of 85, was classified as group II. The remaining branches, which diverge from another node with a bootstrap value of 59, were assigned as the group III. OR types from each of the three groups are listed in the table. **(B)** Visualization of olfactory sensory neurons in sensilla basiconica (ba) and sensilla trichodea (tr) in tissue sections from locust antennae using Orco as a marker. The cluster of OSNs is delineated by white dish lines indicating a small group of Orco positive cells in sensilla trichodea and a large cluster of Orco positive cells in sensilla basiconica.

**FIGURE 2:**
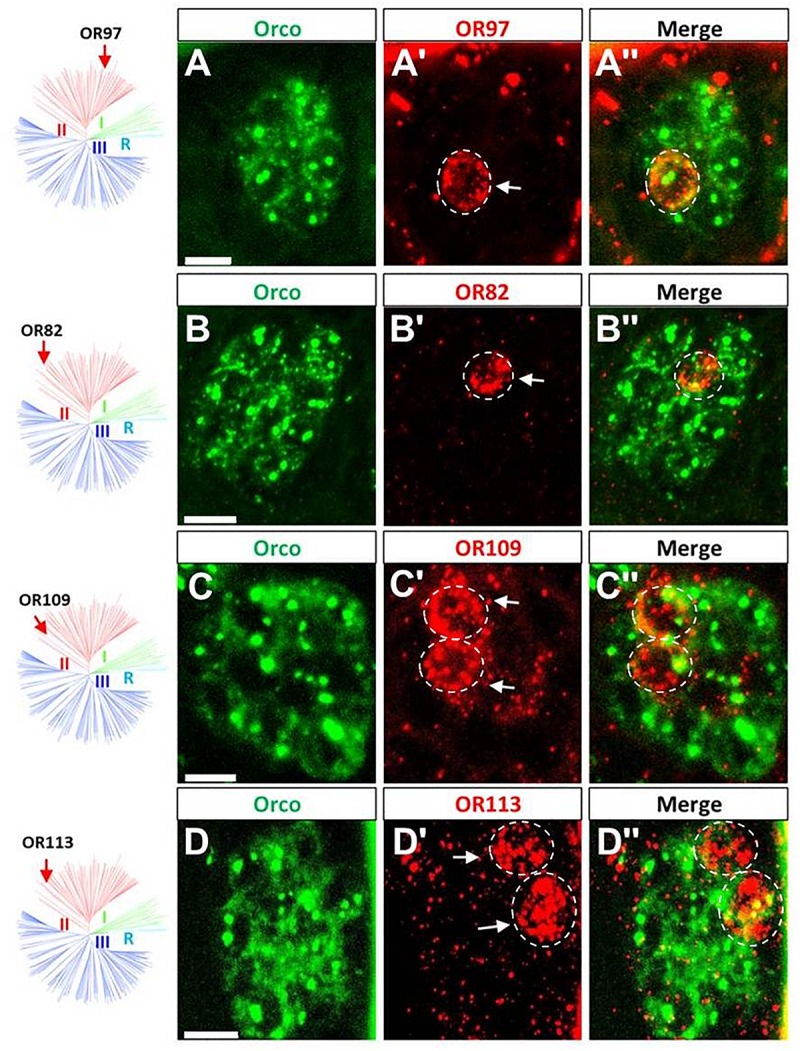
The majority of group II ORs are specifically expressed in sensilla basiconica. **(A–D”)** The Orco- and distinct OR-expressing cells were visualized by means of two-color FISH using specific antisense riboprobes labeled in Bio (green fluorescence) and DIG (red fluorescence), respectively. The four ORs (OR82, OR97, OR109, and OR113) are shown as representatives of group II ORs that are specifically expressed in sensilla basiconica. The white dash circle highlights the cell that concomitantly expresses a defined OR type and Orco. More examples are shown in the supporting data. Distinct OR cells are indicated by white arrows. Scale bars, 10 μm.

**FIGURE 3:**
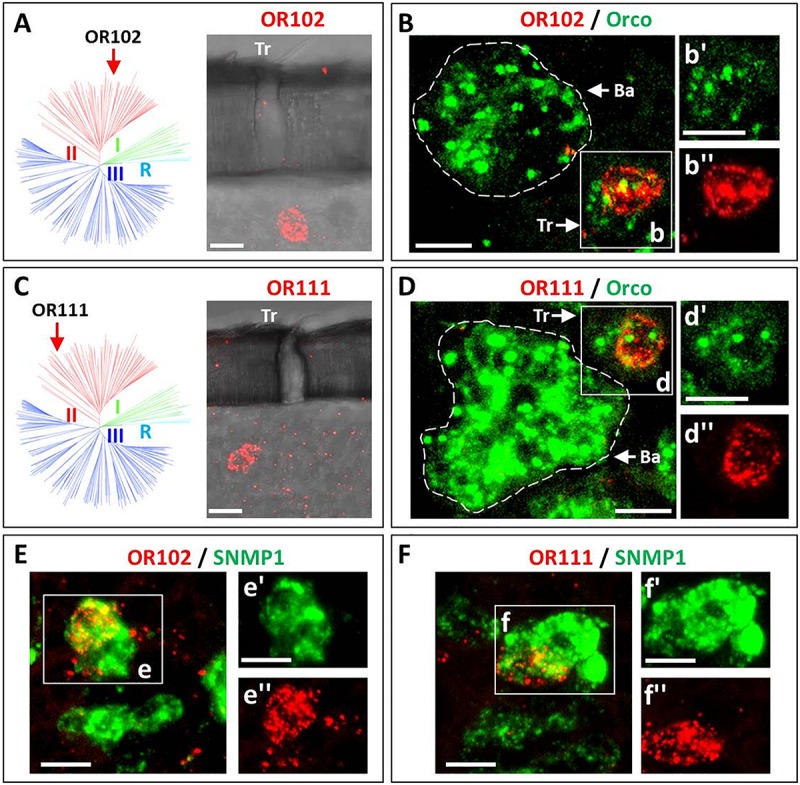
Two ORs from group II are specifically expressed in sensilla trichodea. Cells expressing distinct OR types as well as Orco or SNMP1 were visualized by means of two-color FISH using specific antisense riboprobes labeled in either DIG (OR, red fluorescence) or Bio (Orco and SNMP1, green fluorescence). **(A–D)** The expression of OR102 and OR111 in cells housed in sensilla trichodea is visualized. As indicated in A (OR102) and in C (OR111) labeled cells are located under trichoid (Tr) sensillar hairs. In panel **B,D** co-localization of the two trichoid OR types with Orco are depicted. Discernable basiconic sensilla (Ba) and trichoid sensilla (Tr) cell clusters are indicated by white dash circles and white boxes **(B,D)**, respectively. The insets panel **B’–D”** depict the separate fluorescence channels of panels **B,D**. The white dash circle highlights the cell that concomitantly expresses the defined OR type and Orco. **(E,F)** The co-expression of OR102 and OR111 with SNMP1 is documented. In the insects panels **E’–F”** the separate fluorescence channels of the white boxed areas in panels **E,F** are depicted. The double labeled areas are indicated by white dash circles. Scale bars, 10 μm.

The endogenous OR repertoire is supposed to determine the chemosensory profile of individual sensilla types. For comprehensive analyses of a sensilla-specific expression of the various OR types, we simplified the previously generated phylogenetic tree of locust ORs (Pregitzer et al., 2017) into three groups, namely I, II, and III (Figure 1A). Tissue sections from antennae of adult locusts were analyzed by two color fluorescence *in situ* hybridization (FISH) experiments. Riboprobes labeled by Biotin for the olfactory receptor co-receptor (Orco), a marker for insect OSNs, were employed to visualize the ensemble of all sensory neurons in s. basiconica or s. trichodea, an example is depicted in Figure 1B indicating a large cluster of Orco-positive below a s. basiconica and a small cluster of Orco-positive cells below a s. trichodea. In addition, riboprobes labeled by Digoxigenin (DIG) were used to visualize cells expressing individual OR types. Subsequently, we set out to evaluate almost all OR types (33 ORs) from group II (38 ORs); the results of these experiments are presented in Figure 2 and Supplementary Figure S1. In our analyses, the green labeling of giant OSN clusters, which are indicative of s. basiconica, could be reproducibly visualized, thus allowing to unequivocally determine the sensillum type. We found that most of the examined OR types from group II (31 out of 33) were found to be expressed in s. basiconica. In most of the examined antennal tissue sections only a single labeled cell was observed within the large cell cluster of s. basiconica (Figures 2A,B), however, in a few cases we detected more than one labeled cell in the cell cluster of s. basiconica (Figures 2C,D). This phenomenon was studied in greater detail (see further below). Two receptor types from group II, OR102 and OR111, were found to be expressed in cells housed in trichoid sensilla (Figures 3A,C). Trichoid sensilla comprise a very small set of cells, which make it distinguishable from s. basiconica (Figures 3B,D); for both OR types only a single stained cell was visualized within s. trichodea. This finding, together with the previously identified OR3 from group I, implies that s. trichodea seem to comprise a very limited number of OR types. In *Drosophila melanogaster* and many moth species cells of the trichoid sensilla are characterized by the “sensory neuron membrane protein 1” (SNMP1), a marker of pheromone responsive neurons (Benton et al., 2007; Forstner et al., 2008; Gomez-Diaz et al., 2016; Pregitzer et al., 2017). To evaluate whether the group II receptors OR102 and OR111 may be co-expressed with SNMP1 we performed FISH experiments utilizing riboprobe for each receptor type and a specific riboprobe for SNMP1 labeled by Biotin. As shown in Figures 3E,F the labeling clearly overlapped indicating that the two receptors OR102 and OR111 are in fact co-expressed with SNMP1.

**FIGURE 4:**
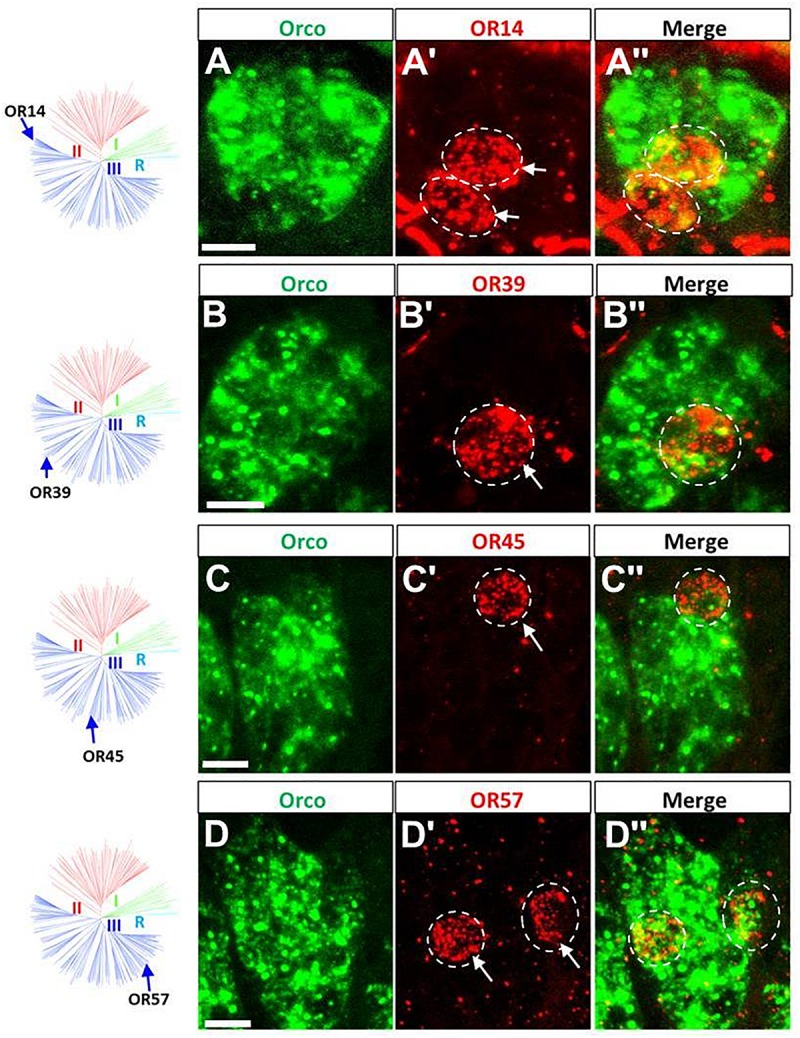
ORs from group III are specifically expressed in sensilla basiconica. The schematic locust OR phylogenetic tree is adapted from Pregitzer et al., 2017 in which R group means Orco rooting group, group I corresponds to the previously classified “b-OR group” and group II and group III are divided based on the branches segregation. The four distinct groups are differentially colored. **(A–D”)** Cells expressing Orco- and distinct OR types were visualized by means of two-color FISH using specific antisense riboprobes labeled in Bio (green fluorescence) and DIG (red fluorescence), respectively. Four OR types (OR14, OR39, OR45, and OR57) are shown as representatives of group III (blue branches) that are specifically expressed in sensilla basiconica. More examples are shown in the supporting data. Distinct OR-positive cells are indicated by white arrows. The white dash circle highlights the cell that concomitantly expresses the defined OR type and Orco. Scale bars, 10 μm.

Subsequently, the question in which sensillum type the receptors from group III may be expressed was approached by two-color FISH experiments as described above. Since group III comprises a large set of OR members, we analyzed as much as 42 receptor types from this group. The localization of some ORs from group III and Orco are depicted in Figure 4 and Supplementary Figure S2; the results indicate that all the examined receptor types from this group were selectively expressed in s. basiconica. Interestingly, also for some of the receptor types from group III more than one labeled cell was observed in the large cell cluster of s. basiconica (Figures 4A,D). For none of the OR types from group III we observe any labeling of cells in s. trichodea. Together, the results of the present and previous studies concerning the sensilla specific expression of OR types from different phylogenetic groups are summarized in Table 1. Out of the total number of 83 examined ORs from the three groups, 80 OR types could be clearly assigned to s. basiconica while only three OR types (OR3, OR102, and OR111) to s. trichodea.

**TABLE 1:** Summary of the sensilla specific expression for 83 receptor types.

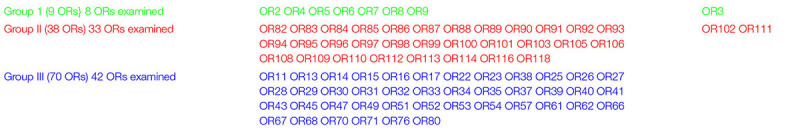

*The specific expression of ORs in sensilla basiconica or in sensilla trichodea was determined by the two-color FISH experiments. Among the 83 analyzed OR types, only 3 ORs namely OR3, OR102 and OR111 were found to be selectively expressed in sensilla trichodea, while all other OR types were expressed in sensilla basiconica.*

### Arrangement of Cells Expressing Distinct Receptor Types in Sensilla Trichodea

**FIGURE 5:**
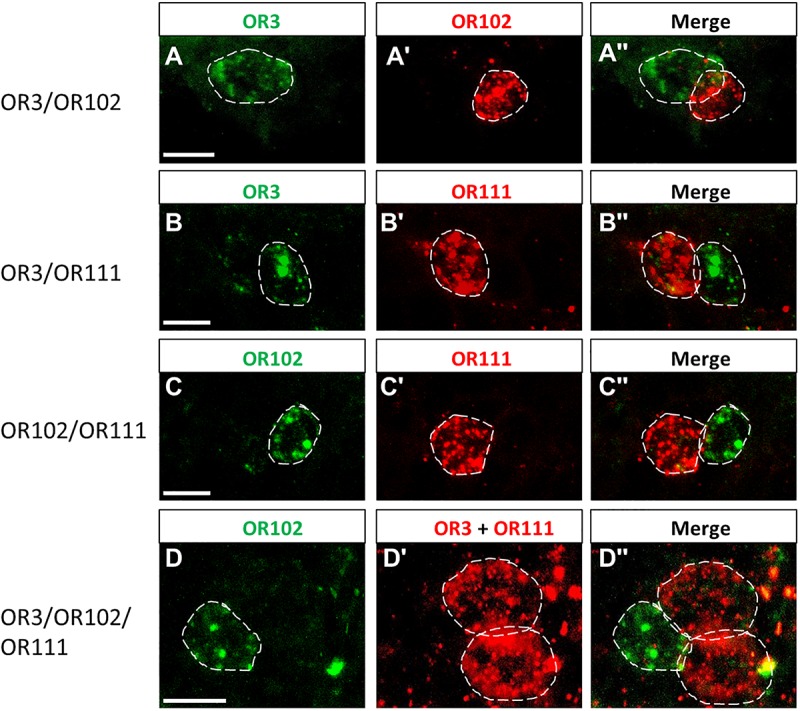
Cells expressing distinct OR types are adjacently located in the trichoid sensilla. **(A–D”)** The relative position of cells in the trichoid sensilla which express distinct OR types was visualized by means of two-color FISH using specific antisense riboprobes labeled in DIG (red fluorescence) or Bio (green fluorescence). White dash circles outline the position of cells expressing a distinct OR type. Note in panel **D** two red-labeled cells were visualized due to the usage DIG-labeled riboprobes for both OR3 and OR111. Scale bar, 10 μm.

Extending the analysis of expression profiles for various OR types may allow evaluating a possibly stereotypical organization of cells expressing certain OR pairs in the cell assembly of an individual sensillum. First, we explored s. trichodea, which have a relatively simple cellular architecture (1–3 OSNs) with a very limited number of receptor types. We pinpointed the relative localization of the three identified OR types focusing on either the expression of two identified receptors types or the expression of all three identified types in a given sensillum. To address this issue we performed two-color FISH experiments with combinations of individual riboprobes for OR3, OR102, and OR111, which are either DIG- or Biotin-labeled; the emerged results are depicted in Figure 5. We have found that in s. trichodea each of the three receptors was strictly expressed in only one single cell; no indication for any over representation was observed (Figure 3). Using riboprobes for only two receptor types, we obtained a labeling of cells located side by side (Figures 5A–C”), confirming the genuine existence of such hypothesized OR combinations. Moreover, using riboprobes for all three receptor types, in rare cases we faithfully visualized a cluster of three labeled cells that are located adjacently; indicative for an expression all three OR types in a given sensillum (Figures 5D–D”). Such co-existence of the three receptor types within a single trichoid sensillum was additionally confirmed by conducting single color FISH experiment using DIG-labeled riboprobes for all these three receptors (Supplementary Figure S3). Based on these observations it is conceivable that the cell assembly in s. trichodea adopt multiple combinations of receptor types, in which the three OR types (OR3, OR102, and OR111) readily participate in forming a variety of functional combinations.

### In Sensilla Basiconica Certain OR Types Are Expressed in Multiple Cells

**FIGURE 6:**
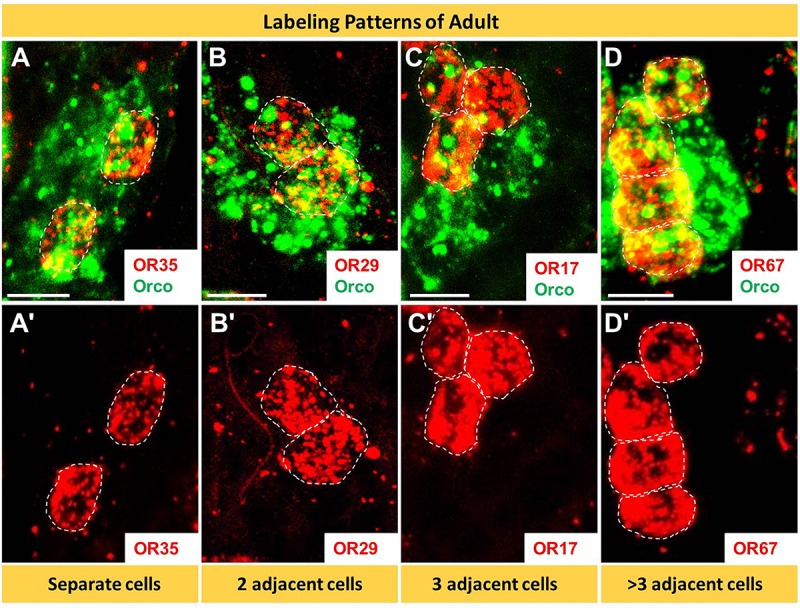
In basiconic sensilla distinct OR types are expressed in several cells. The cells expressing distinct OR types and Orco were visualized by means of two-color FISH using specific antisense riboprobes. **(A–D’)** The analyses of 10 μm optical stacks revealed four different patterns of OR expression; ranged from solitary cells to multicellular clusters. White dash circles outline cell expressing the distinct OR type. Scale bars, 10 μm.

In the multicellular assembly of s. basiconica certain OR types were found to be expressed in more than one cell (see Figures 2, 3). To address this issue of “over representation” for certain OR types in more detail, we have quantified six representative OR types from group I to III, namely OR8, OR17, OR29, OR35, OR67, and OR110. Firstly, we assessed their expression profile by performing comprehensive two-color FISH analyses using differentially labeled riboprobes targeting the selected OR types and Orco, respectively. As a result, for the multicellular expression of a given receptor type in s. basiconica as much as four labeling patterns emerged (Figure 6): receptors could be expressed in 2 cells (OR29, OR35), 3 cells (OR17) or even 4 cells (OR67). Upon a closer inspection, we found that in most cases cells expressing the same OR type were adjacently distributed, forming a cluster of cells. Scrutinizing antennal sections, we have observed a variety of clusters comprising 2 cells, 3 cells and even more than 3 cells (Figure 6).

**FIGURE 7:**
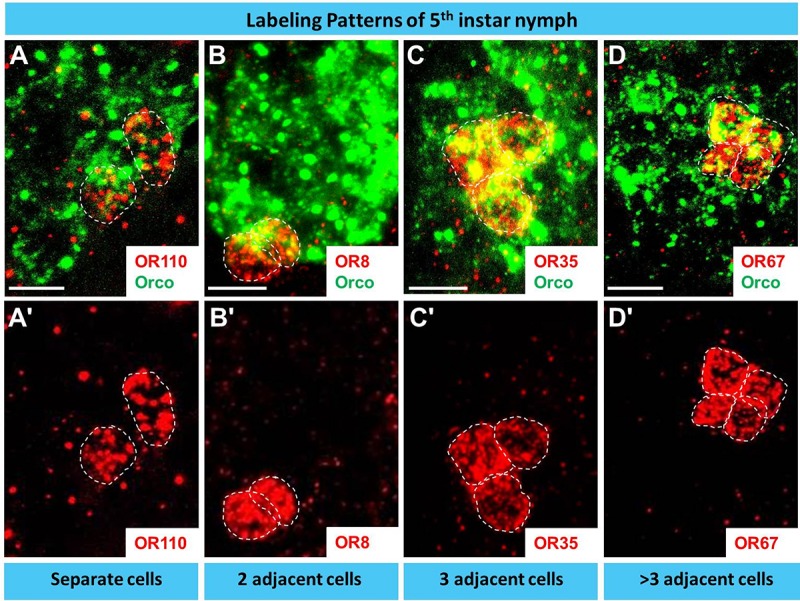
During late development (5th instar nymph) distinct OR types are expressed in multiple cells basiconic sensilla. In **A–D’** cells expressing a distinct OR type and Orco were visualized by means of two-color FISH using specific antisense riboprobes. The analyses of 10 μm optical stacks from sections of 5th instar nymph antennae revealed four different patterns of OR expression, similar to adults. White dash circles outline distinct OR cells. Scale bar, 10 μm.

**FIGURE 8:**
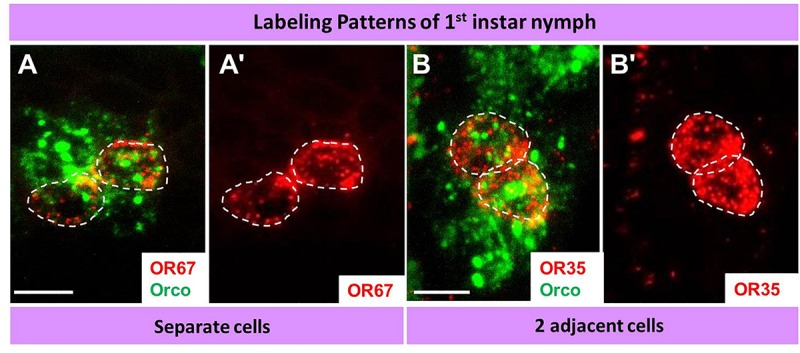
During early development (1st instar nymph) a distinct OR type is only expressed in relatively few cells in basiconic sensilla. In **A–B’** cells expressing distinct OR types and Orco were visualized by means of two-color FISH using specific antisense riboprobes. The analyses of 10 μm optical stacks from sections of 1st instar nymph antennae revealed only two patterns OR expression: solitary cells or cell pairs. White dash circles outline distinct OR cells. Scale bars, 10 μm.

**FIGURE 9:**
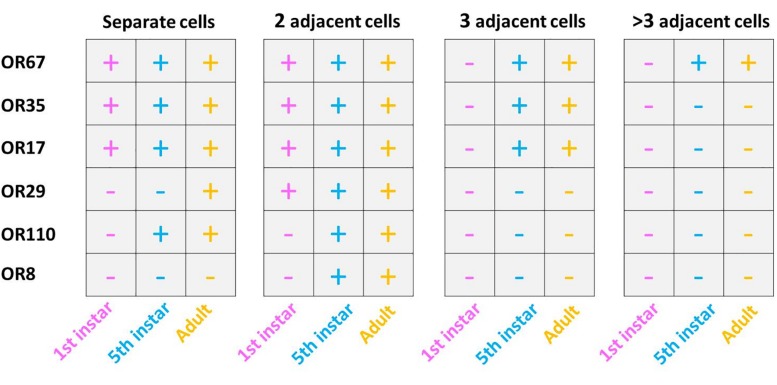
Developmental dependent expression patterns of SgreOR’s in basiconic sensilla OSNs. Expression patterns in three developmental stages of six analyzed ORs. Observed cells and labeling patterns are indicated by a “**+**,” whereas the absence of such is indicated by “**–**.” The results for this figure are based upon three independent FISH experiments.

The question arises whether the number and spatial arrangement of cells expressing a distinct receptor type is established from the very beginning or whether those cells expressing the same receptor type emerge gradually in the course of development. Desert locusts undergo a hemimetabolous life cycle and grow continuously by successive molts. In a next step, we analyzed the topographic expression of these receptors in the antennae of 5th instar nymphs, the final nymph stage, when the overall body and antennal size is close to the adult stage. At this nymph stage, we found the four types of expression pattern, which closely resembled that of adults (Figure 7). Subsequently, we assessed antennae from 1st instar nymphs, which significantly differ in size from 5th instar nymphs. The results of the FISH assays are documented in Figure 8 indicating that at this stage in the multicellular assembly of s. basiconica only single cells or maximal 2 cell clusters (in the case of OR67 and OR35) exist, whereas there was no indication for 3 or more cell clusters. The results concerning the dynamics of OR cell populations are summarized in Figure 9, which suggests that a representation of distinct OR types by several cells in individual basiconic sensilla gradually occurs in the course of locust development.

## Discussion

The results of the present study may contribute toward an understanding of the observation that morphologically identifiable sensilla on the antennae of the desert locust *Schistocerca gregaria* comprise OSNs, which respond to different categories of chemical signals (Ochieng’ and Hansson, 1999). In order to elucidate decisive molecular elements, which may underlay the sensilla-specific responsiveness of these cells, a large repertoire of odorant receptor types was assessed concerning the sensilla-specific expression patterns. Probing more than 80 OR types revealed that the vast majority of OR types were selectively expressed in sensilla basiconica, whereas only three OR types were identified in sensilla trichodea (Figures 2–4 and Table 1). The large difference correlates to some extent with the much higher number of s. basiconica on the antennal surface (more than 1000) as well as the higher number of OSNs (20–50) per sensillum basiconicum (Ochieng et al., 1998).

The finding that in the antennae of *S. gregaria* apparently only very few OR types are expressed in s. trichodea is interesting in view of the recent observation that in *Locusta migratoria* up to 16 different trichoid neuron types were identified based on different odorant response spectra (Cui et al., 2011). This discrepancy could either be due to species-specific expansion of trichoid receptors, the effect of different auxiliary proteins in the sensilla, like odorant binding proteins or biotransformation enzymes, but possibly also due to additional OR types still to be discovered in *S. gregaria*. In this context it is worthwhile to point out that in a few cases each of the three neurons in a trichoid sensillum expressed one of the trichoid-specific receptors, OR3, OR102, and OR111, whereas in most cases two of the three receptors were found to be expressed in a trichoid sensillum (Figure 4). This observation could either be due to the fact that a considerable proportion of trichoid sensilla comprise only two chemosensory cells (Ochieng et al., 1998; Cui et al., 2011) or that additional relevant receptor types exist; nevertheless, the current results would argue against the existence of a large number of additional trichoid-specific OR types in *S. gregaria*. Based on the currently available data it remains elusive whether in s. trichodea from *S. gregaria*, which house 1–3 OSNs (Ochieng et al., 1998), the cells stereotypically express one of the three specific OR types or whether distinct combinations of OR types are expressed in different types of trichoid sensilla. In this context, it is interesting to note that in flies and moths a stereotypic expression pattern of OR types in defined sensilla has been observed, i.e., a given OR type was only expressed in a distinct type of sensillum and always distinct matching OR types were expressed in the two cells of the sensillum (Hallem et al., 2004; Krieger et al., 2009).

The approaches to explore the principles of OR expression in the multicellular assembly of s. basiconica with 20–50 sensory neurons (Ochieng et al., 1998), revealed that also in this case most of the OR types seem to be expressed in one or few of the multiple cells. However, it is interesting to note that certain OR types were found to be expressed in up to four adjacent cells (Figures 6- 9 and Supplementary Figure S4). The reason and functional implication for this surprising observation is unclear. The finding that the expression of certain OR types in several cells was not observed in early stage does imply that it emerges gradually in the course of development. Such a developmental process would be in line with the hemimetabolous life cycle of *S. gregaria*, i.e., while with each molt the body size and number of antennal segments increases, probably also the number of cells in each segment rises (Ochieng et al., 1998). Whether cells determined to express a distinct receptor type proliferate during this early phase of development or whether the appropriate OR types are expressed in newborn cells is currently unclear. Moreover, it might be possible that distinct sensory cues encountered during early life induce the “over-representation” of certain OR types in multicellular assembly of s. basiconica. Though effects of external sensory cues and the developmental of sensory neurons remain elusive, it is interesting to note that exposure to certain olfactory cues can alter the volume of glomeruli (Devaud et al., 2003; Arenas et al., 2012) or the activity of higher-order sensory neurons (Sachse et al., 2007).

Extensive research over the last decades have uncovered several characteristic features of the locust olfactory system, which significantly deviate from the generally assumed design for insect olfactory systems, mainly based on studies on moths and flies. An obvious difference can be ascribed to the antennal lobe, the first-order olfactory center of the insect brain; in contrast to most insect species, the antennal lobe of locusts display a microglomerular organization with more than a thousand very small glomeruli per lobe (Ernst et al., 1977; Ignell et al., 2001). The processing of olfactory information in this unique neuronal network has been an intense area of research for many years (Anton and Hansson, 1996; Laurent, 2002). However, very little is known how the sensory information from the antennae is conveyed into the neuronal network of the lobe. This aspect is of particular interest in view of the relatively small repertoire of OR types, i.e., the number of OR-specific OSN populations are much smaller than the number of glomeruli. Although deviations from the 1 OR - 1 glomerulus rule has been observed in some other species, in locust the discrepancy is particular striking. This raises fundamental questions concerning the wiring pattern of an OR-specific OSN population.

It is conceivable that there are subpopulations of OR-specific OSNs, e.g., due to the position in the antennae, each subpopulation projecting to its microglomeruli; alternatively, the whole population of OR-specific OSNs could project to multiple microglomeruli. In this context, it is interesting to note, that in locusts the axon from an individual OSN branches and in this way can target several different glomerular structures in the antennal lobe (Hansson et al., 1996). Interestingly, using a transgenic approach such an axonal projection pattern was also visualized for OSNs in the vomeronasal system of rodents, which project their axons to six to ten glomeruli in the accessory olfactory bulb (Del Punta et al., 2002). The knowledge about the genes encoding ORs and their sensilla-specific expression may allow to employ similar approaches in locusts to label OR-specific OSNs in the antennae and visualize the targeting pattern of their axons in the antennal lobe.

## Data Availability

All datasets generated for this study are included in the manuscript and or Supplementary Files.

## Author Contributions

XJ, HB, and PP: current study conception and results interpretation. XJ and PP: experiment conduction, data acquisition, and preliminary manuscript composition. PP and HB: refinement and approval of final manuscript.

## Conflict of Interest Statement

The authors declare that the research was conducted in the absence of any commercial or financial relationships that could be construed as a potential conflict of interest.
