# Supplementary material for: Sensilla-Specific Expression of Odorant Receptors in the Desert Locust Schistocerca gregaria
Source: Front Physiol. 2019 Aug 22;10:1052. doi: 10.3389/fphys.2019.01052 (PMC6714038; doi:10.3389/fphys.2019.01052)
Supplement: Supplementary file 1 [file Data_Sheet_1.PDF]

Supporting Data

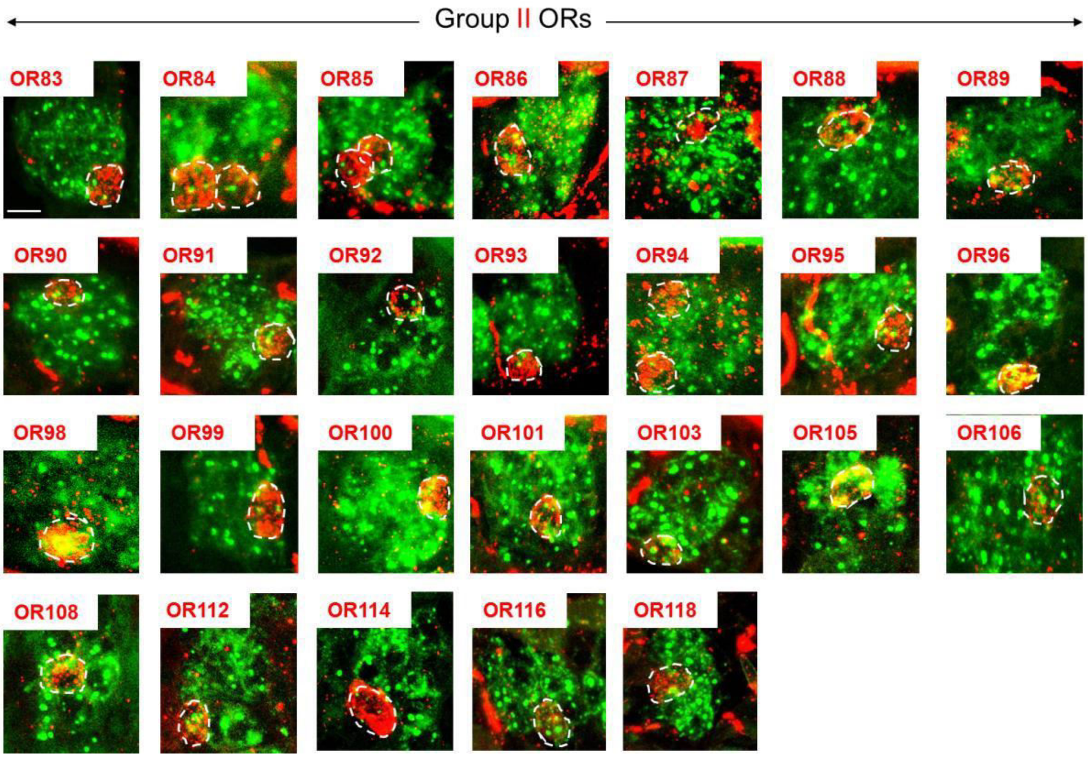

Figure S1

Assembly of 26 additional group II ORs expressed in s. basiconica. Staining of distinct OR types (in red) and Orco (in green) were visualized by means of two-color FISH using DIG- and BIO-labeled riboprobes, respectively. OR-positive cells are delineated by white dash lines. Scale bar, 10 µm.

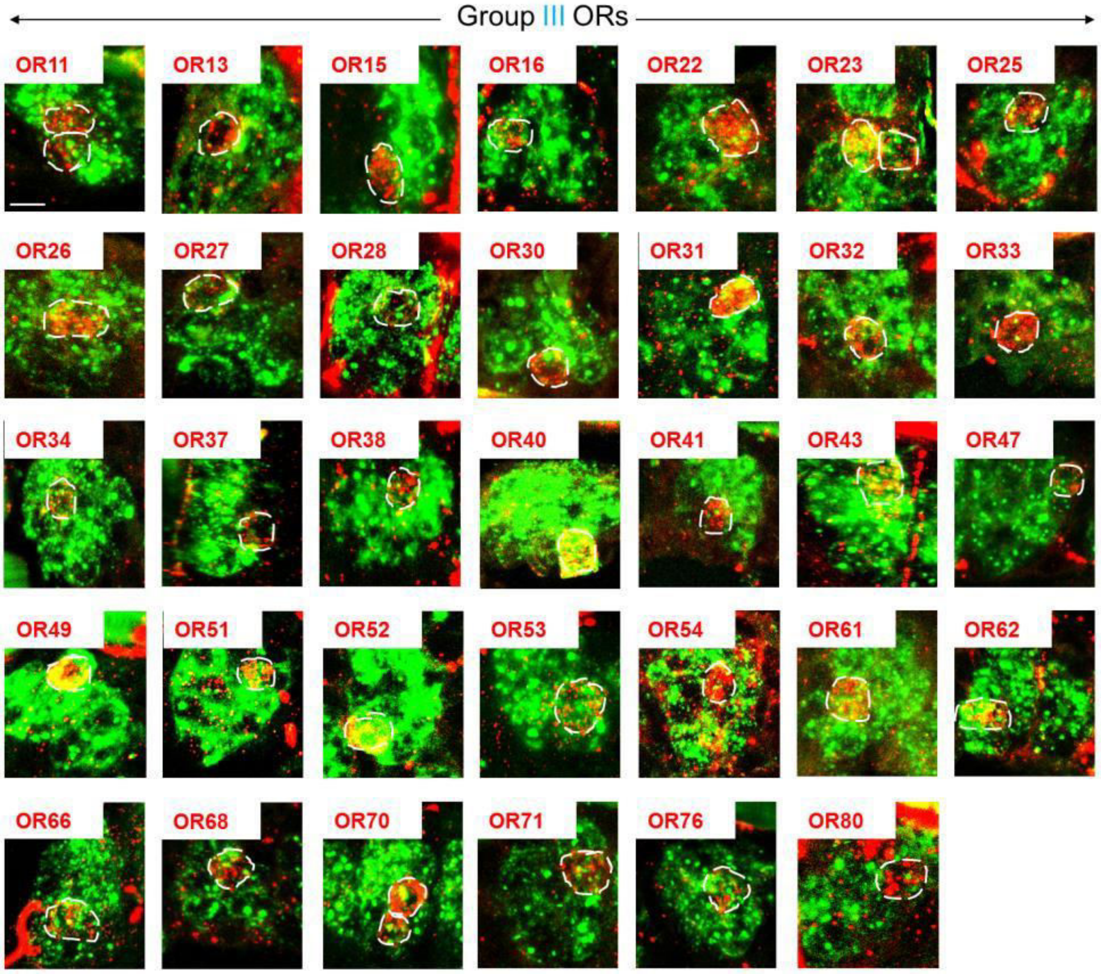

Fig. S2

34 additional group III ORs expressed in s. basiconica. Staining for distinct OR types (in red) and Orco (in green) was visualized by means of two-color FISH using DIG- and BIO-labeled riboprobes, respectively. OR-positive cells are delineated by white dash lines. Scale bar, 10 µm.

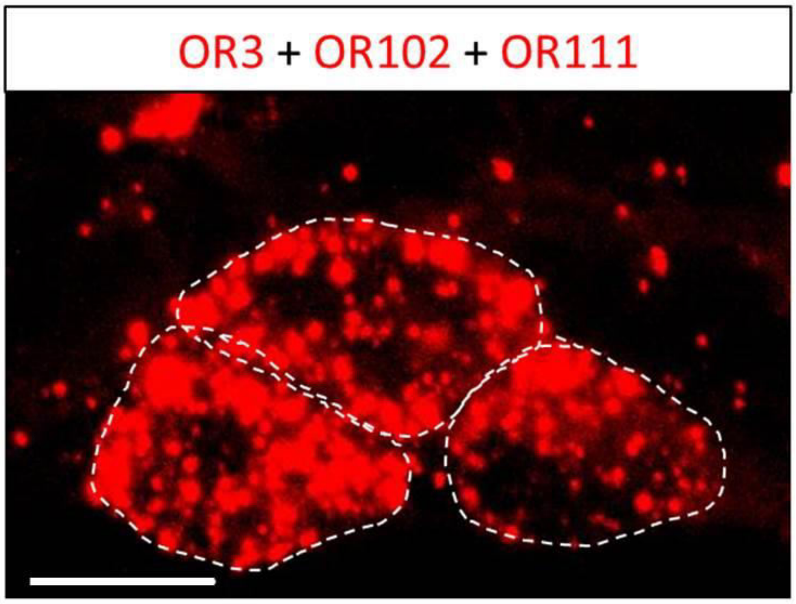

Figure S3
Co-localization of cells expressing three trichoid sensilla OR types

The cells expressing three distinct OR types were visualized by means of single FISH using a mixture of antisense riboprobes labeled in DIG (red fluorescence). White dash circle outlines three discernable OR cells. Scale bar, 10 μm.

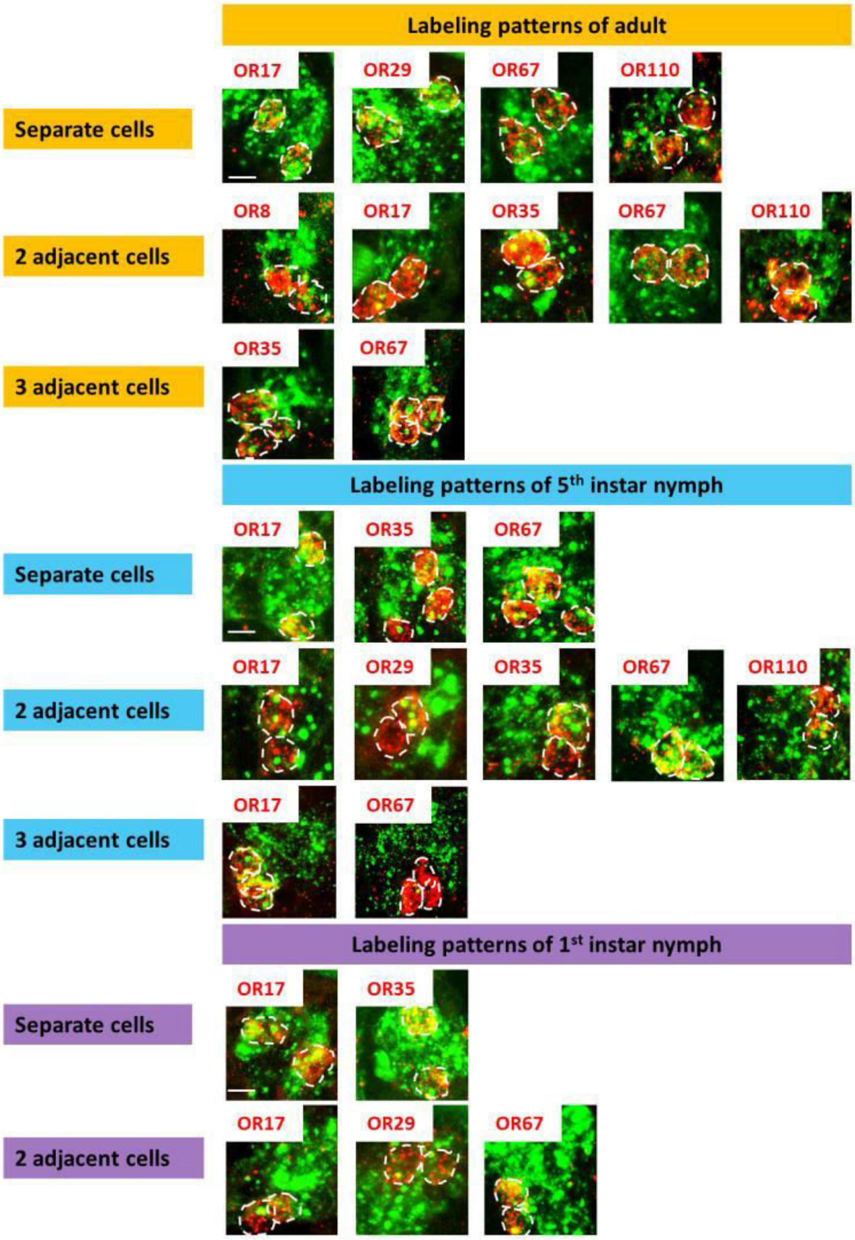

Fig. S4

Additional examples of OR labeling patterns in s. basiconica at three different developmental stages. Labeling for distinct OR types (in red) and Orco (in green) were visualized by means of two-color FISH using DIG- and BIO-labeled riboprobes, respectively. OR-positive cells are delineated by white dash lines. Scale bar, 10 µm.
